# Cellular internalization mechanism of novel Raman probes designed for plant cells†

**DOI:** 10.1039/d0cb00128g

**Published:** 2020-08-20

**Authors:** Keiko Midorikawa, Kousuke Tsuchiya, Simon Sau Yin Law, Yu Miyagi, Takuya Asai, Takanori Iino, Yasuyuki Ozeki, Yutaka Kodama, Keiji Numata

**Affiliations:** Biomacromolecules Research Team, RIKEN Center for Sustainable Resource Science 2-1 Hirosawa Wako Saitama 351-0198 Japan keiji.numata@riken.jp kosuke.tsuchiya@riken.jp; Department of Material Chemistry, Graduate School of Engineering, Kyoto University Kyoto-Daigaku-Katsura Nishikyo-ku Kyoto 615-8510 Japan; Department of Electrical Engineering and Information Systems, The University of Tokyo Tokyo 113-8656 Japan; Center for Bioscience Research and Education, Utsunomiya University Tochigi 321-5805 Japan kodama@cc.utsunomiya-u.ac.jp

## Abstract

Diphenylacetylene derivatives containing different polymeric components, poly(l-lysine) (pLys) or tetra(ethylene glycol) (TEG) were designed as novel Raman imaging probes with high Raman sensitivity and low cytotoxicity in living plant cells. The pLys-conjugated probe is internalized *via* an endocytosis-dependent pathway, whereas TEG-conjugated probe most likely induces direct penetration into the plant cells.

## Abstract 2

Diphenyl acetylene derivatives containing various polymeric components have been designed as new Raman imaging probes. These are taken up by plant cells *via* different pathways, and the internalization of exogenous molecules can be visualized.

## Graphical abstract

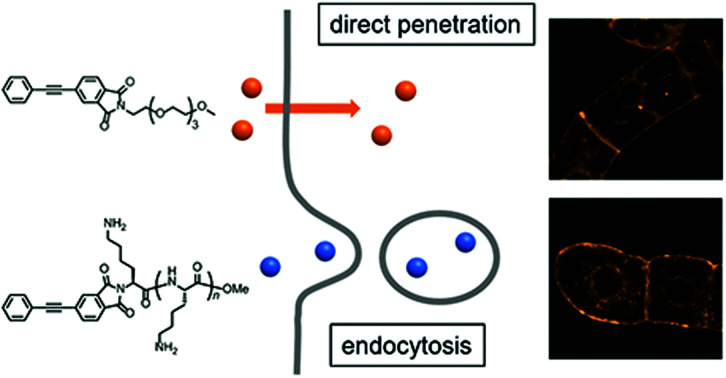

Plant cells synthesize many more secondary metabolites than animal cells, many of which are useful to humans.^1,2^ Therefore, much effort has been made to stabilize production and increase yields for these metabolites by plant biotechnology. Recently, an intracellular delivery system using a complex with a polymer carrier has emerged as a tool that enables cellular internalization of various bioactive molecules.^3,4^ These molecules contribute to the improvement of production efficiency of metabolite with genetic modification. In order to further improve the delivery of materials it is important to elucidate the internalization mechanisms that remain unknown. Thus, the tools are needed to visualize the process of target molecule internalization in living cells. Site-selective staining using fluorescent probes has been developed to visualize how biomolecules are localized in living cells using fluorescence microscopy.^5^ However, following the target molecules themselves is difficult in fluorescence microscopy using conventional probe dyes unless the target molecules are chemically labelled with a fluorescent probe *via in vivo* reactions. On the other hand, Raman microscopy can detect a vibration attributed to a specific chemical structure within target molecules and can be applied to observe molecular behaviour in living cells without invasion or staining. The alkyne group is the best candidate as a Raman probe for modifying target molecules because it produces a specific peak in a silent region of the Raman spectrum,^6^ which enables detection of the probe in molecular crowding situations found in living cells. Furthermore, since the acetylene moiety is a bioorthogonal functional group with an inert nature to chemical reactions *in vivo*, it has the advantage that it is less susceptible to non-specific interactions and intracellular reactions. Therefore, the fluorescent probe needs to be designed in consideration of non-specific interactions *in vivo* and unexpected chemical reactions, whereas the Raman probe can be applied to a wide range of targets. In recent years, the introduction of alkynyl groups into low-molecular-weight compounds, such as drugs and metabolites, has been reported for visualization *in vivo*.^7,8^

Notably, the metabolites of interest must be independently modified with a Raman-detectable moiety for visualization in the previous examples. To date, analysis using Raman microscopy has been applied effectively to both animal and plant cells.^9–12^ Therefore, a novel Raman-based monitoring system would be highly beneficial for the visualization of various phenomena associated with molecular translocation in living plant cells. However, in general, Raman spectromicroscopic analysis has lower detection sensitivity than fluorescence probe imaging. To enhance the sensitivity, Raman probes require a chemical structure with a stronger Raman signal intensity, such as diphenylacetylene moieties. The drawbacks of these structures are hydrophobic, water-insoluble, cytotoxic, and poorly permeable into plant cells.^13,14^ Hence, we have overcome these problems by adding a hydrophilic and highly biocompatible polymeric carrier to diphenylacetylene, which shows strong Raman intensity. Besides, further functionalization enables such the polymeric component-fused Raman probes to be utilized for delivery of various target molecules into a cell, as we have reported that the polymeric carriers can be easily combined with arbitrary functional molecules *via* the thiol–ene reaction when the polymeric carriers are modified with a maleimide group.^3^

Herein, we developed novel Raman probes that enable us to monitor the active translocation of target molecules in plant cells with high Raman sensitivity and low cytotoxicity to plant cells. We combined diphenylacetylene with two types of polymeric components, poly(l-lysine) (pLys) and poly(ethylene glycol) (PEG) of different lengths. The pLys and PEG components, which are both hydrophilic but membrane-interactive or physiologically inert, respectively, are often used as part of carriers to deliver exogenous molecules. The two types of conjugated materials were found to function as efficient Raman probes in plant cells with different uptake mechanisms depending on the polymeric components. Furthermore, to improve the sensitivity, we used stimulated Raman scattering (SRS) microscopy.^15,16^ The current system combining the novel Raman probes and SRS imaging allows us to analyse molecular behaviours in live cells at a practical level.

Amine-terminated pLys (*n* = 4–8, the average DP is 6), tetra(ethylene glycol) (TEG), and PEG (*n* = 24) were conjugated with 4-phenylethynylphthalic anhydride to obtain the Raman probes, PhC≡CPh-pLys (**1**), PhC≡CPh-TEG (**2**), and PhC≡CPh-PEG (**3**), respectively (Fig. 1a). The peptide pLys was selected as membrane-permeable component according to the screening study of cell penetrating peptides (CPPs) for plant cells.^17^ The CPPs designated for animal cells showed different internalization behaviour in plant cells, and Lys-containing peptides showed a relatively high internalization efficiency in plant cells.

**Fig. 1:**
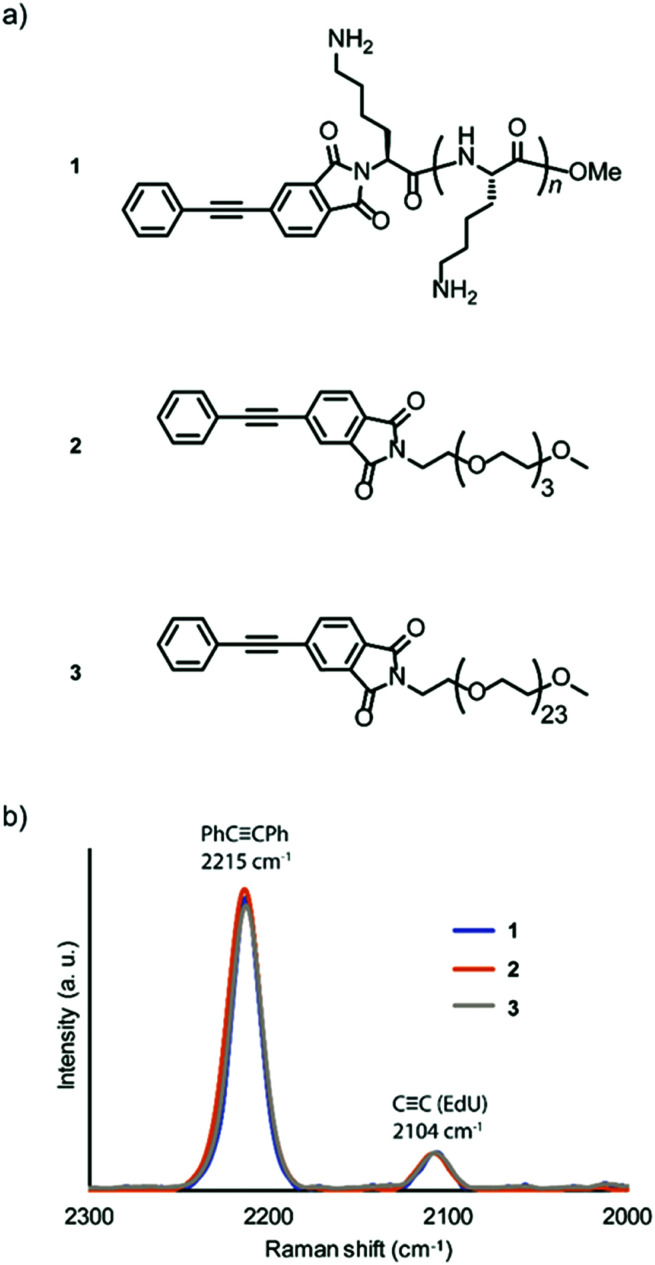
Relative Raman intensity of new probes. (a) Chemical structure of Raman probes. PhC≡CPh-pLys (**1**), PhC≡CPh-TEG (**2**), PhC≡CPh-PEG (**3**). (b) Comparison of Raman intensity/shift between three types of probes. The spectrum was normalized to the Raman intensity of EdU.

The chemical structures of the probes were confirmed by ^1^H NMR spectroscopy and matrix-assisted laser time-of-flight (MALDI-TOF) mass spectrometry (Fig. S1–S6, ESI†). The alkyne-tagged thymidine analog 5-ethynyl-2′-deoxyuridine (EdU) was used as an internal standard for the probe solutions,^18^ and the obtained spectra were normalized to compare the Raman intensities. All three probes showed a strong peak in the same Raman shift region (2215 cm^−1^). Additionally, we confirmed that the intensities of the three probes were similar (Fig. 1b), with higher intensity than the standard ethynyl compound EdU.

Although both EdU and diphenylacetylene have alkynyl groups, diphenylacetylene shows higher Raman intensity because of the aromatic rings at both ends, which leads to the enhancement of Raman scattering. We confirmed that this enhanced Raman effect was maintained when the polymeric component was combined. Then, in order to investigate whether these probes are applicable to plant cells, we examined the internalization of the probes by Raman microscopy using tobacco bright yellow 2 (BY-2) cells and *Arabidopsis thaliana*. The BY-2 cell line is the most widely used plant cell culture.^19^ After the BY-2 cells were incubated in a probe-containing medium, the peak at 2215 cm^−1^ assignable to the diphenylacetylene moiety was detected within the cytoplasm of cells in the Raman spectra (Fig. 2a and b).

**Fig. 2:**
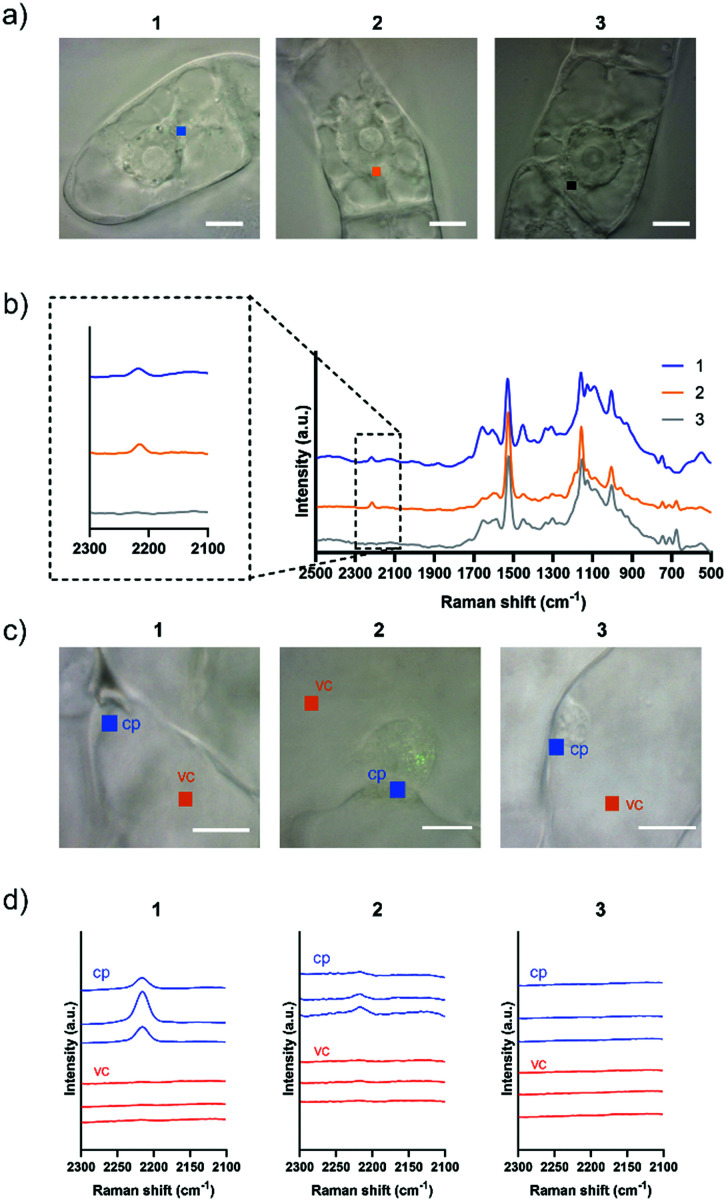
Internalization of Raman probes into BY-2 cells and *Arabidopsis thaliana* leaf cells. (a) Squares indicate irradiated points in the cytoplasm in BY-2 cells (1 μm × 1 μm in size). Scale bars are 10 μm. (b) Averaged Raman spectra in the cytoplasm of BY-2 cells were obtained after 5 h treatment with 100 μM **1**, **2**, or **3**. Diphenylacetylene shows a Raman peak at 2215 cm^−1^. (c) Squares indicate irradiated points in the cytoplasm (cp) and vacuole (vc) in a *var2-1* leaf cells (1 μm × 1 μm in size). Scale bars are 10 μm. (d) Raman spectra in a *var2-1* leaf cells were obtained after 5 to 8 h treatment with 100 μM **1**, **2**, or **3**. Three spectra were obtained at different positions in each cite.

The internalization of **1** and **2** into BY-2 cells was observed, whereas no Raman peak for the diphenylacetylene moiety was detected in the cells treated with **3**. We confirmed that the signal from Raman probe **1** and **2** was detected at multiple points in one BY-2 cell, mainly within the cytoplasm region (Fig. S7 and S8, ESI†). These results indicate that the pLys and TEG components function as effective carriers to internalize the hydrophobic diphenylacetylene moiety, which hardly dissolves in an aqueous buffer. In contrast, the long PEG chain hampered the internalization of the probe, probably because a long PEG chain shows minimal nonspecific interaction with biomolecules, including membrane lipids, due to its large exclusion volume.^20^ Similarly, we carried out an experiment using *Arabidopsis thaliana*, which is a model plant. In the experiment with leaf cells, we used *var2-1* mutant plant having variegated leaves to suppress autofluorescence derived from chlorophyll in leaves (Fig. S9, ESI†).^21–23^ The *var2-1* mutant plant has mutations in the subunits of the thylakoid-localized FtsH complex that are likely involved in chloroplast biosynthesis and development.^24,25^ The leaves were dipped in the 100 μM probe solution, then washed and observed with a Raman microscope. As a result, internalization into cytoplasm was confirmed for **1** and **2**. On the other hand, the Raman peak of the diphenylacetylene moiety was not detected in the cell treated with **3** (Fig. 2c and d). Similar results were obtained with root cells (Fig. S10, ESI†). These results indicate that the pLys and TEG components can be applied *in planta*.

For Raman probes **1** and **2**, which showed internalization into plant cells, we carried out a cell viability assay in BY-2 cells (Fig. S11, ESI†). Notably, the cytotoxicity of **1** was higher than that of **2** at the concentration used in this experiment (100 μM), and TEG-modified probe **2** showed negligible cytotoxicity, which is associated with the different interactions of **1** and **2** with biological membranes. Then, we further investigated the intracellular uptake mechanism of the probe using BY-2 cells in which the intracellular structure is easier to observe. The membrane was stained with an endocytic tracer FM4-64, and the cells were observed by confocal laser scanning microscopy (CLSM) (Fig. 3a). The biological membrane in BY-2 cells was entirely stained in all the cells treated with three probes. In particular, only the cells treated with **1** showed characteristic vesicle structures with a size less than 500 nm stained with FM4-64 in the cytoplasmic area in addition to the other biological membrane (Fig. 3b). We assume that these vesicles are endosomes formed by endocytosis. This result suggests that both probes **1** and **2** are taken into the cells, but their internalization pathways are different.

**Fig. 3:**
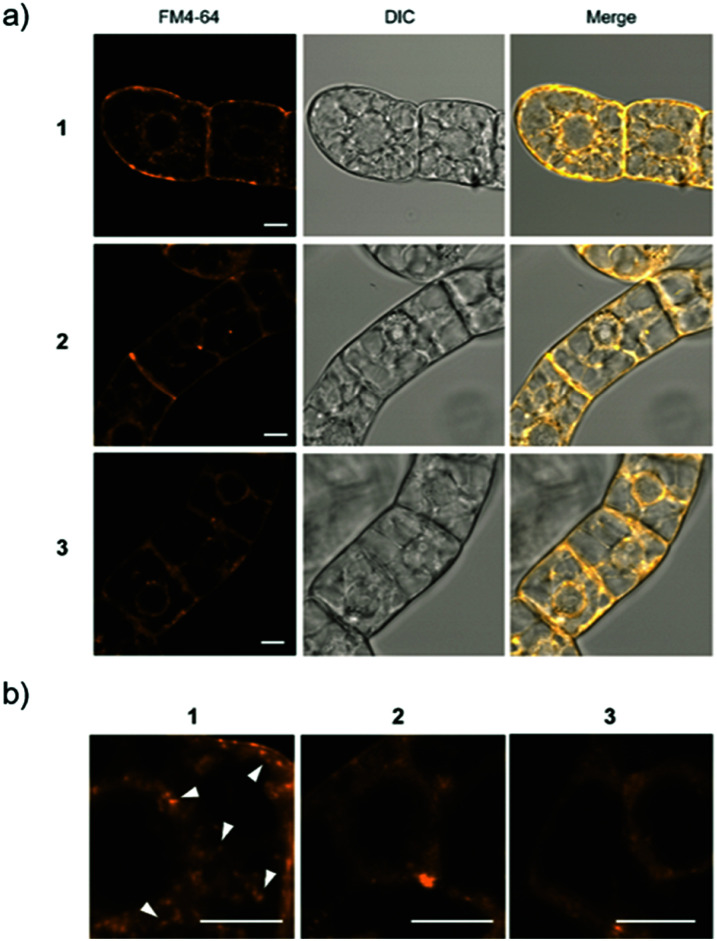
CSLM imaging of BY-2 cells. (a) Cells treated with each Raman probe were stained with FM4-64 (yellow). (b) Expanded CLSM images for the cells treated with **1**, **2**, or **3**. Arrowheads show vesicles with a size less than 500 nm induced by internalization of **1**. Scale bars are 10 μm.

To investigate whether intracellular internalization of **1** is dependent on endocytosis, we performed two kinds of endocytosis inhibition assays. First, we performed low-temperature treatment to stop the major endocytosis pathway in the cells.^26^ BY-2 cells were treated with **1** or **2** and incubated at 4 °C for 5 h. Then, the probe signal in the cytoplasm was detected by Raman microscopy. As a result, the probe signal was detected in the cells treated with **2** but not with **1** (Fig. 4a and Fig. S12, ESI†).

**Fig. 4:**
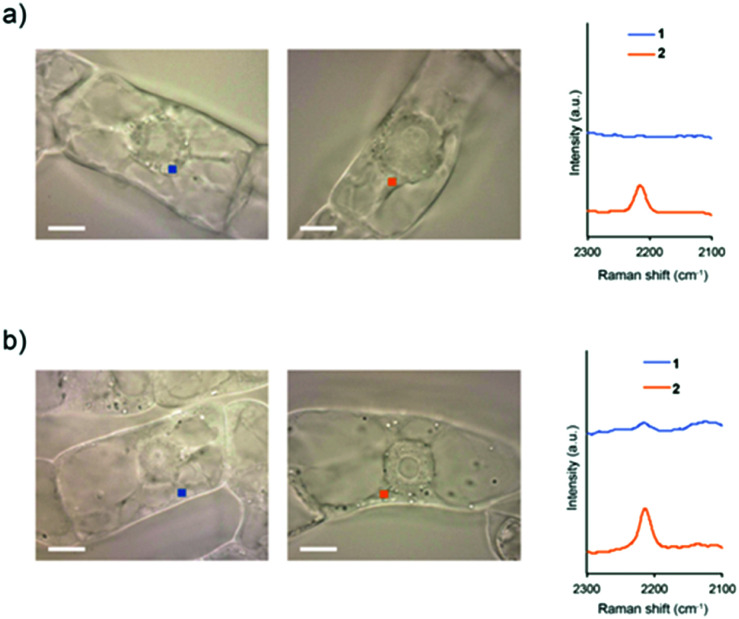
Raman spectra of BY-2 cells in the cytoplasm. (a) The cells were treated with **1** or **2** at 4 °C. (b) The cells were treated with **1** or **2** in the presence of Wortmannin. The spectra were obtained after 5 h of treatment with 100 μM **1** or **2**. The cytoplasm area in BY-2 cells (1 μm × 1 μm in size) used for obtaining the averaged Raman spectra are presented by squares in the images. Scale bars are 10 μm.

Subsequently, the same experiment was conducted by treatment with Wortmannin, an endocytosis inhibitor. Wortmannin is a specific inhibitor of the phosphatidylinositol (PI) 3-kinase that has been shown to block endocytosis in mammalian cells.^27^ It also has been shown to be active in plant cells, where it inhibits protein sorting to the vacuole through action on both the PI 3- and PI 4-kinases.^28^ In the cells treated with Wortmannin, the signal of **2** was detected, whereas the signal of **1** was faintly observed (Fig. 4b and Fig. S13, ESI†). Compared with untreated cells, both low-temperature and Wortmannin treatments provided a significant decrease in the Raman intensity at 2215 cm^−1^ in the BY-2 cells treated with **1** (Fig. S14, ESI†). From these results, it was clear that endocytosis is a dominant pathway for the internalization of **1** into BY-2 cells. In contrast, because **2** was efficiently internalized into the cells even under endocytosis-inhibited conditions, it is assumed that the internalization of **2** is most likely *via* an energy-independent pathway such as direct penetration.

Hitherto, transmission electron microscopy and atomic force microscopy analyzes have shown that gold nanoparticles coated with pLys or PEG are encapsulated in vesicles and taken up to cells.^29,30^ Nevertheless, the probe containing the PEG component was not applicable to plant cells. It is considered that the PEG-probe caused aggregate formation, so that it could not pass through the cell wall meshwork structure.

Finally, to investigate the intracellular localization of these probes in detail, we performed SRS imaging analysis because of its rapid visualization for the distribution of the Raman probes in cells with high sensitivity.^15,16^ We observed BY-2 cells treated with the Raman probes by SRS microscopy. SRS images were obtained at three Raman shifts: 2190, 2235, and 2215 cm^−1^. The peak intensity at 2215 cm^−1^ was used to visualize the cells after subtracting the background of the average between 2190 and 2235 cm^−1^. The obtained images revealed that the three types of Raman probes show different intracellular internalization (Fig. 5).

**Fig. 5:**
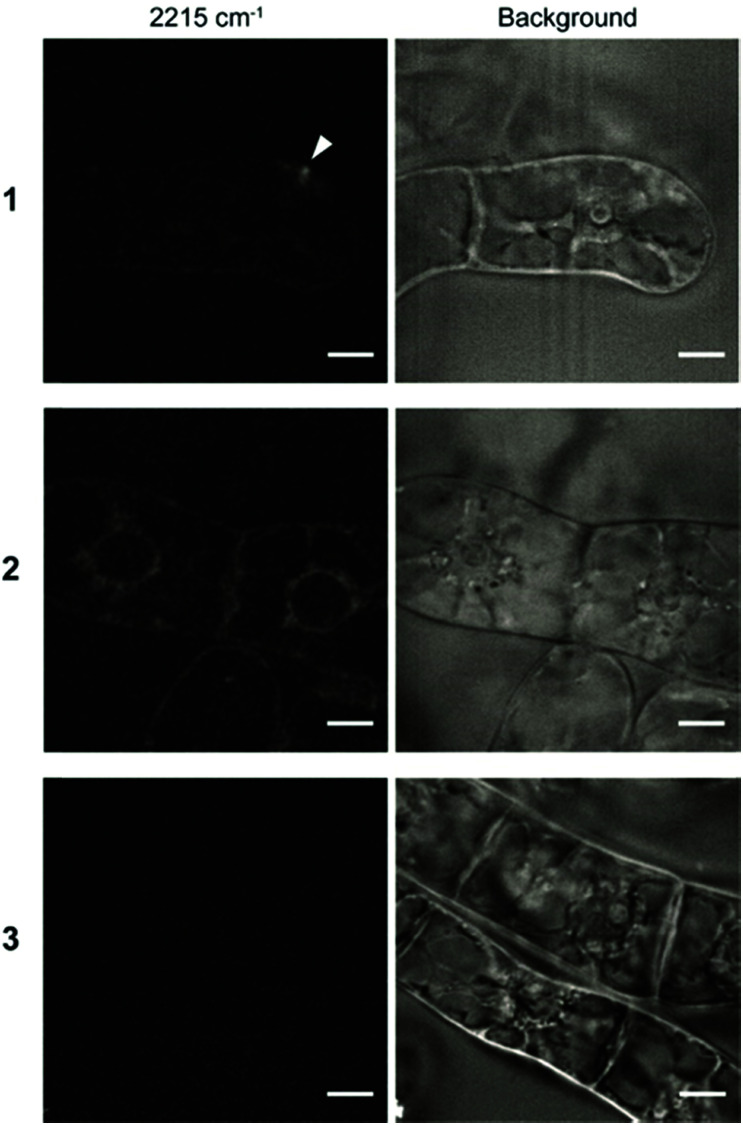
SRS imaging of BY-2 cells treated with Raman probes. BY-2 cells were incubated for 8 h in solution containing Raman probes **1–3** (100 μM). The images at 2215 cm^−1^ (left) show the diphenylacetylene moiety of the Raman probe. Background (right) is the images using average intensities of 2190 and 2235 cm^−1^. The arrowhead shows the putative endosome observed in the cell treated with **1**. Scale bars are 10 μm.

In the cells treated with **1**, a strong signal was detected at a specific site in the cells by SRS imaging. Since we assume that the internalization of **1** proceeds *via* endocytosis, it is considered that the accumulation site was captured by SRS imaging. Meanwhile, the signal of **2** was detected throughout the cytoplasm. Unlike **1**, **2** showed efficient intracellular uptake by direct penetration. The energy-independent direct penetration pathway probably allows easier accumulation in cells than the energy-dependent endosomal pathway.^31^ In addition, probe **3** was not detected in any of the cells, which is consistent with the results of Raman microscopy and CLSM shown in Fig. 2 and 3. The result revealed that the structural difference of the polymeric components (pLys or TEG) resulted in the different internalization and localization which play an important role in delivery of exogenous materials into plant cells.

In summary, we demonstrated that the novel alkyne compounds consisting of a diphenylacetylene moiety with a high signal intensity and hydrophilic polymeric components, pLys and TEG. Interestingly, these Raman probes exhibit different intracellular internalization mechanisms depending on the added macromolecular components. The pLys component is considered to interact with biological membranes and dominantly induce endocytosis. In contrast, since TEG shows little interaction with membranes, the probe with the TEG component was proven to be most likely internalized into the cells *via* direct penetration with negligible cytotoxicity. Based on the findings obtained from this study, practical Raman probes showing high Raman sensitivity, high permeability in plant cells, and low cytotoxicity can be developed by combining diphenylacetylene and specific polymeric components. As an ongoing study, by attaching a reactive functional group to diphenylacetylene, such as a maleimide group, it is possible to bind target cargos, including plasmid DNA, enzyme proteins, and other physiologically active molecules to be delivered intracellularly. The modification with a functional group to the diphenylacetylene moiety is expected to enable tracking of the target cargos by the Raman probe with maintaining the function of pLys or TEG as a membrane-permeable carrier. Visualization of molecules using such Raman probes in plant cells will allow us to obtain deep insights for biological research on the internalization process into plant cells during material delivery.

## Conflicts of interest

The authors have no conflicts to declare.

## Supplementary Material

CB-001-D0CB00128G-s001
