# Supplementary material for: Cellular internalization mechanism of novel Raman probes designed for plant cells
Source: RSC Chem Biol. 2020 Aug 20;1(4):204–8. doi: 10.1039/d0cb00128g (PMC8341950; doi:10.1039/d0cb00128g)
Supplement: CB-001-D0CB00128G-s001 [file CB-001-D0CB00128G-s001.pdf]

Electronic Supplementary Material (ESI) for RSC Chemical Biology.
This journal is © The Royal Society of Chemistry 2020

Electronic Supplementary Information

Cellular Internalization Mechanism of Novel Raman Probes Designed for Plant Cells

Keiko Midorikawa[†a], Kousuke Tsuchiya*[†a,d], Simon Sau Yin Law[a], Yu Miyagi[a], Takuya Asai[b], Takanori Iino[b], Yasuyuki Ozeki[b], Yutaka Kodama*[a,c], and Keiji Numata*[a,d]

[a] Biomacromolecules Research Team, RIKEN Center for Sustainable Resource Science, 2-1 Hirosawa, Wako, Saitama 351-0198, Japan.

[b] Department of Electrical Engineering and Information Systems, The University of Tokyo, Tokyo 113-8656, Japan

[c] Center for Bioscience Research and Education, Utsunomiya University, Tochigi 321-5805, Japan

[d] Department of Material Chemistry, Graduate School of Engineering, Kyoto University, Kyoto-Daigaku-Katsura, Nishikyo-ku, Kyoto 615-8510, Japan.

[†] These authors are co-first author.

Table of contents

Experimental details 2

Supplementary figures (Fig. S1-S14) 7

References 22

Experimental

Materials and Analytical Methods.

Poly[N_ε-(*tert*-butoxycarbonyloxy)-L-lysine] [P(Boc-Lys)] was prepared by papain-catalyzed polymerization of N_ε-(*tert*-butoxycarbonyloxy)-L-lysine ethyl ester hydrochloride in aqueous buffer as previously reported.[1] All the other materials were purchased from Watanabe Chemical Industries, Ltd. (Hiroshima, Japan), Tokyo Chemical Industry Co., Ltd. (Tokyo, Japan), or Kanto Chemical Co., Inc. (Tokyo, Japan) and used as received. ^{1}H (500 MHz) and ^{13}C (125 MHz) NMR spectra were recorded on a VARIAN NMR spectrometer (Varian Medical Systems, Palo Alto, CA). IR spectra were acquired on a Shimadzu IRPrestige-21 spectrophotometer (Shimadzu Corporation, Kyoto, Japan) with a MIRacle A single reflection ATR unit using a Ge prism. MALDI-TOF MS spectra were taken on an ultrafleXtreme MALDI-TOF spectrophotometer (Bruker Daltonics, Billerica, MA) operating in reflection mode at an accelerating voltage of 15 kV. The sample was dissolved in water/acetonitrile (0.8 mg mL^{-1}) containing 0.1% trifluoroacetic acid (TFA), mixed with a solution of α-cyano-4-hydroxycinnamic acid (CHCA) in water/acetonitrile (10 mg mL^{-1}), and deposited on an MTP 384 ground steel BC target plate. Analytical reverse-phase high-performance liquid chromatography (RP-HPLC) was performed on an HPLC system consisting of auto sampler AS-2055, gradient pump PU2089, column oven CO-4060, UV/Vis detector UV-4075, and quaternary gradient pump PU-2089 Plus (JASCO, Tokyo, Japan). A Reprosil Gold 120 C18 column (150 x 4 mm, 5 μm) was used with a flow rate of 1 mL min^{-1} at 50°C. The mobile phase consisted of acetonitrile (eluent A), Milli-Q water (eluent B), and Milli-Q water supplemented with 0.1 v/v% TFA (eluent C). The sample solution was injected and eluted by a mixed mobile phase with a linear gradient from 88% A, 2% B, and 10% C to 64% A, 26% B, and 10% C over 24 min.

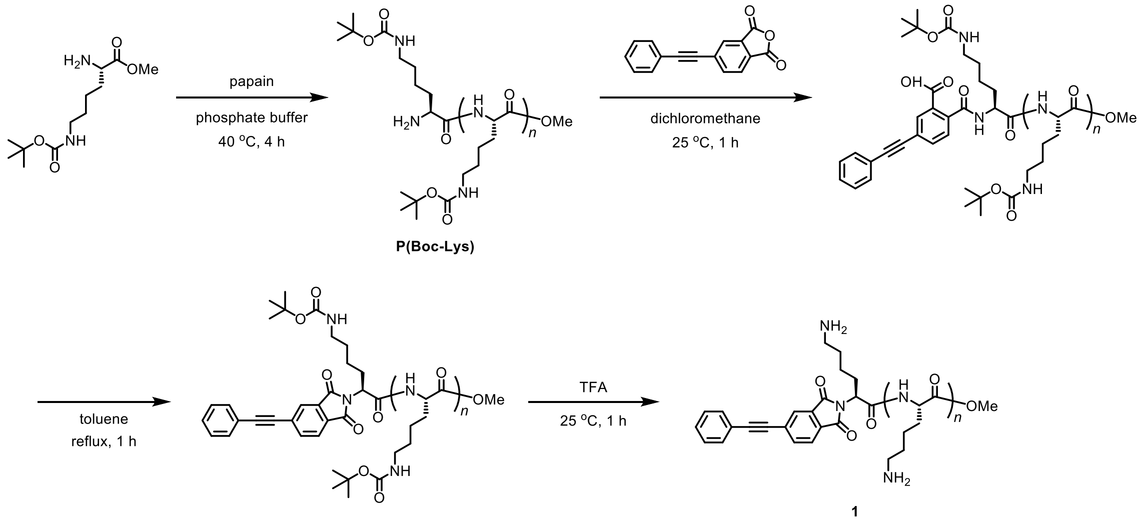

Synthesis of PhC≡CPh-pLys (1)

P(Boc-Lys) (420.6 mg, 0.3 mmol) and 4-phenylethynylphthalic anhydride (74.47 g, 0.3 mmol) were added to a flask with a stirrer bar under nitrogen at 25 °C. After CH_2Cl_2 (6 mL) was added, the solution was stirred for 1 h at 25°C and then added to an excess amount of hexane. The precipitate was gathered by filtration. The amide carboxylic acid derivative was obtained as a white solid in 87% yield (431.6 mg, 0.262 mmol). Then, the solid (237 mg, 0.144 mmol) was dissolved in toluene (2.8 mL) under nitrogen at 25°C. The solution was refluxed at 130°C for 1 h, and the solvent was removed under reduced pressure. The crude product was purified by aluminum oxide column chromatography with $CHCl_3$/MeOH mixture as the eluent. The imide derivative was obtained as a yellow solid. For the deprotection of Boc groups in the side chain, the obtained yellow solid (90 mg, 0.178 mmol) was placed in a flask. Trifluoroacetic acid (0.272 mL, 3.55 mmol) was added to this solution and stirred at 25°C. After 24 h, the solvent was removed under reduced pressure. The obtained viscous solid was dispersed in water and lyophilized. The title compound was obtained as a white solid in 48% yield (71.3 mg, 0.069 mmol). ^{1}H NMR (500 MHz, D_2O): δ 1.15-1.44 (m, $-CH^*(CH_2C\mathit{H}_2CH_2CH_2NH_2-)-$), 1.45-1.86 (m, $-CH^*(CH_2CH_2C\mathit{H}_2CH_2NH_2-)-$), 2.00-2.10 (m, $-CH^*(C\mathit{H}_2CH_2CH_2CH_2NH_2-)-$), 2.75-2.93 (m, $-C\mathit{H}_2NH_2$), 3.63 (m, $-OC\mathit{H}_3$), 4.10-4.27 (m, $-NC\mathit{H}^*(CH_2-)CO-$), 4.60-4.85 (m, $-NHC\mathit{H}^*(CH_2-)CO-$, $-CH_2N\mathit{H}_2$), 7.36 (m, 3H, Ar), 7.50 (m, 2H, Ar), 7.79 (t, 1H, Ar), 7.89 (m, 2H, Ar). ^{13}C NMR (125 MHz, D_2O): δ 21.9-22.4, 26.1-26.3, 29.5-30.6, 38.9-39.1, 52.6-54.1, 87.6, 93.9, 121.4, 123.9, 126.1, 128.7, 129.6, 129.7, 129.7, 131.3, 131.7, 137.8, 168.9, 173.3-174.0. IR (neat): 3071, 2941, 1778, 1661, 1634, 1518, 1435, 1339, 1140, 999, 841, 799, 723 cm^{-1}.

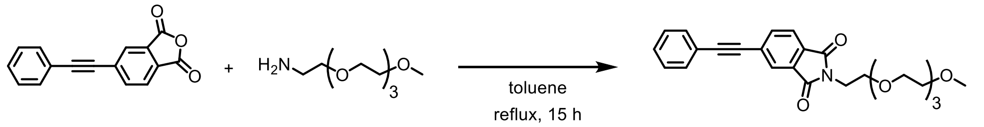

2

Synthesis of PhC≡CPh-TEG (2)

3, 6, 9, 12-Tetraoxatridecanamine (100 mg, 0.48 mmol) and 4-phenylethynylphthalic anhydride (119.8 g, 0.48 mmol) were added to a flask with a stirrer bar under nitrogen at 25°C. After toluene (9.65 mL) was added, the solution was stirred for 1 h and refluxed at 130°C for 24 h. The solution was concentrated by using a rotary evaporator. The white crude solid was purified by silica gel column chromatography with ethyl acetate as the eluent (R_f = 0.5). The title compound was obtained in 90% yield (342.6 mg, 0.434 mmol). ^{1}H NMR (500 MHz, $CDCl_3$): δ 3.45-3.65 (m, 95H, –OCH_3, –OCH_2CH_2O–), 3.72 (t, J = 5, 2H, –NCH_2CH$_2$O–), 3.87 (t, J = 5, 2H, –NCH$_2$CH_2O–), 7.36 (m, 3H, Ar), 7.51 (m, 2H, Ar), 7.78 (t, 2H, Ar), 7.91 (m, 1H, Ar). ^{13}C NMR (125 MHz, $CDCl_3$): δ 37.3, 58.8, 67.7, 69.9, 70.4, 71.8, 87.7, 93.7, 122.0, 123.0, 125.9, 128.4, 129.1, 129.3, 130.7, 131.7, 132.3, 136.6, 167.4. IR (neat): 2874, 1773, 1715, 1616, 1495, 1437, 1389, 1354, 1323, 1250, 1192, 1107, 1026, 918, 851, 758, 746 cm^{-1}.

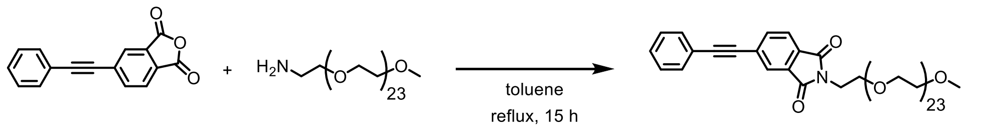

3

Synthesis of PhC≡CPh-PEG (3)

PhCCPh-PEG was synthesized according to the same procedure as PhCCPh-TEG. Amine-terminated poly(ethylene glycol) (PEG, degree of polymerization = 24) was used as the starting material instead of 3, 6, 9, 12-tetraoxatridecanamine. The product was obtained as a white powder with a yield of 80%. ^{1}H NMR (500 MHz, $CDCl_3$): δ 3.49-3.65 (m, 15H, –OCH_3, –OCH_2CH_2O–), 3.69 (t, J = 5, 2H, –NCH_2CH$_2$O–), 3.84 (t, J = 10, 2H, –NCH$_2$CH_2O–), 7.34 (m, 3H, Ar), 7.50 (m, 2H, Ar), 7.77 (t, 2H, Ar), 7.90 (m, 1H, Ar). ^{13}C NMR (125 MHz, $CDCl_3$): δ 37.2, 58.8, 67.7, 69.9, 70.4, 71.8, 87.6, 93.7, 121.9, 123.1, 125.9, 128.4, 129.0, 129.3, 130.6, 131.7, 132.2, 136.6, 167.4. IR (neat): 3071, 2941, 1778, 1661, 1634, 1518, 1435, 1339, 1140, 976, 841, 799, 723 cm^{-1}.

Plant Materials and Growth Conditions.

Suspension-cultured tobacco (*Nicotiana tabacum*) BY-2 cell line (rpc00001) was provided by RIKEN BRC, Japan. The BY-2 cells were grown in Murashige-Skoog (MS) medium supplemented with 3% (w/v) sucrose, 0.2 g L^{-1} KH_2PO_4, 1 mg L^{-1} thiamine·HCl, 0.1 g L^{-1} myo-inositol, and 0.2 mg L^{-1} 2,4-dichlorophenoxyacetic acid (2,4-D), adjusted to pH 5.8. Subculturing was performed once a week. The cultures were kept in the dark at 26°C. Log phase cells (3 or 4 days after culture) were used throughout the study unless otherwise stated.

Arabidopsis thaliana (Col-0) was used as the wild-type. The Arabidopsis *var2-1* mutant was provided by Dr W. Sakamoto (Okayama university).[2] The seeds were sterilized in 70% ethanol for 1 min then in 10% NaClO for 15 min, after which they were rinsed three times with sterilized water to remove the NaClO. The seeds were grown on 1/2 Murashige and Skoog medium (MS Basal Medium, M5519; Sigma-Aldrich, St. Louis, MO) containing 2.5 mM MES, 1% sucrose, and 0.8 % (w/v) agarose. After being sown on the medium, the seeds were stratified for two days in the dark at 4 °C, then grown under constant white light (100 μmol m−2 s−1) at 22 °C. In the experiment, 14-day-old seedlings were used.

Raman Microscopy.

All Raman spectra were obtained on a Jasco NRS-4100 laser Raman spectrometer (JASCO, Tokyo, Japan) with excitation at 532 nm. Samples were placed on a microscope slide (SUPERFROST WHITE, S2441, Matsunami, Osaka, Japan) with a covered slip (C218181, Matsunami). The laser output was focused on the sample (UPLSAPO 100XO, Olympus, Tokyo, Japan). The slit size of the spectrograph was 100 x 8000 μm. The light intensity was 33.8 mW/μm^2. For each spectrum, the measurement was duplicated with an acquisition time of 60 s and averaged.

Preparation of the Raman Probe-Treated Cells.

Each Raman probe was introduced into BY-2 cells. We prepared 10 mM stock solutions of PhC≡CPh−pLys (**1**), PhC≡CPh−TEG (**2**), and PhC≡CPh−PEG (**3**) in DMSO. The cultured cells were incubated in medium containing 100 μM each probe for 5 to 8 h. Cells were rinsed with MS medium three times before the next procedure. To introduce each probe into Arabidopsis leaf or root cells, seedlings were immersed in 100 μM probe solution and incubated at 23° C for 5 to 8 h. It was rinsed twice with distilled water before the observation.

Confocal Laser Scanning Microscopy.

BY-2 cells stained with FM4-64 were observed by confocal laser scanning microscopy (LSM800,

Carl Zeiss, Oberkochen, Germany). After the Raman probe treatment, to stain the plasma membrane, BY-2 cells were immersed in a solution of 20 μM FM4-64 (Thermo Fisher Scientific, Waltham, MA) for 15 min. Cells were rinsed with MS medium before observation.

Endocytosis Inhibition Assay.

In the low-temperature treatment, BY-2 cells were incubated with Raman probes (final concentration: 100 μM) at 4°C in the dark for 5 h. In the inhibitor treatment, BY-2 cells were incubated with a Raman probe (final concentration: 100 μM) and 40 μM wortmannin ((+)-Wortmannin, 230-02341; Wako Pure Chemical Industries, Osaka, Japan) at 26°C in the dark. After the treatments, cells were rinsed with MS medium before observation by Raman microscopy.

Stimulated Raman Scattering (SRS) Imaging of Probe-treated Cells.

The images of the cells were obtained with the SRS imaging system, as previously reported.[3] Briefly, picosecond pump pulses at a wavelength of 843 nm were generated by a Ti:sapphire laser, and wavelength-tunable Stokes pulses at ~1030 nm were generated by an in-house built Yb fiber laser synchronized to the pump laser. The pump and Stokes pulses were combined with a dichroic mirror and sent to a laser scanning SRS microscope, where they were focused by a water immersion objective lens (60×, NA = 1.2) on a sample sandwiched by two cover slips. The optical power of the pump and Stokes pulses were estimated to be ~75 mW and ~55 mW, respectively, at the sample plane. The transmitted pump pulses were detected by a photodiode, and its output was sent to a lock-in amplifier to obtain the SRS signal. SRS images at wavenumbers of 2190 cm^{-1}, 2215 cm^{-1} and 2235 cm^{-1} were acquired successively at a frame rate of 30 frames/sec and averaged over 500 times. The total acquisition time was 50 s. The average image of 2190 and 2235 cm^{-1} were used as a background image. Because no Raman signal was detected at these regions, the background images were obtained as a difference of irradiated laser intensity reflecting the difference of metabolite concentration which absorbs and/or scatters the laser. The average images of 2190 and 2235 cm^{-1} were subtracted from the image of 2215 cm^{-1} as background.

Cell Viability Assay.

The cell viability of BY-2 cells against Raman probes was evaluated by Evans blue (EB) staining. The method was almost the same as in the previous report.[4] For the assay, the Raman probe (final concentration: 100 μM) was incubated with BY-2 cells (OD_{600}: 0.5) at 26°C int he dark. The

incubated BY-2 cells were washed with Milli-Q water and mixed with 50 μg mL^{-1} EB for 10 min. The stained BY-2 cells were washed with Milli-Q water and treated with methanol/SDS solution for 2 h. The lysates were centrifuged, and the supernatants were measured in OD_{600}. As a positive control (100% dead cells), BY-2 cells were incubated for 1 h at 100°C to kill them completely, and then 50 μg mL^{-1} EB was added for 10 min and washed three times.

Hydrodynamic Size and ζ Potential Measurements

The Raman probes were characterized by a ζ potentiometer (Zetasizer Nano-ZS; Malvern Instrument, Ltd., Worcestershire, UK). Each Raman probe solution was prepared to a final concentration of 100 μM using Milli-Q. The ζ potential and ζ deviation of samples were measured three times by a ζ potentiometer, and the average data were obtained using Zetasizer software ver. 7.12 (Malvern Instruments, Ltd.). Dynamic light scattering (DLS) was performed to determine the hydrodynamic diameter with a ζ nanosizer (Zetasizer software ver. 7.12) using a 633 nm He-Ne laser at 25°C with a backscatter detection angle of 173°.

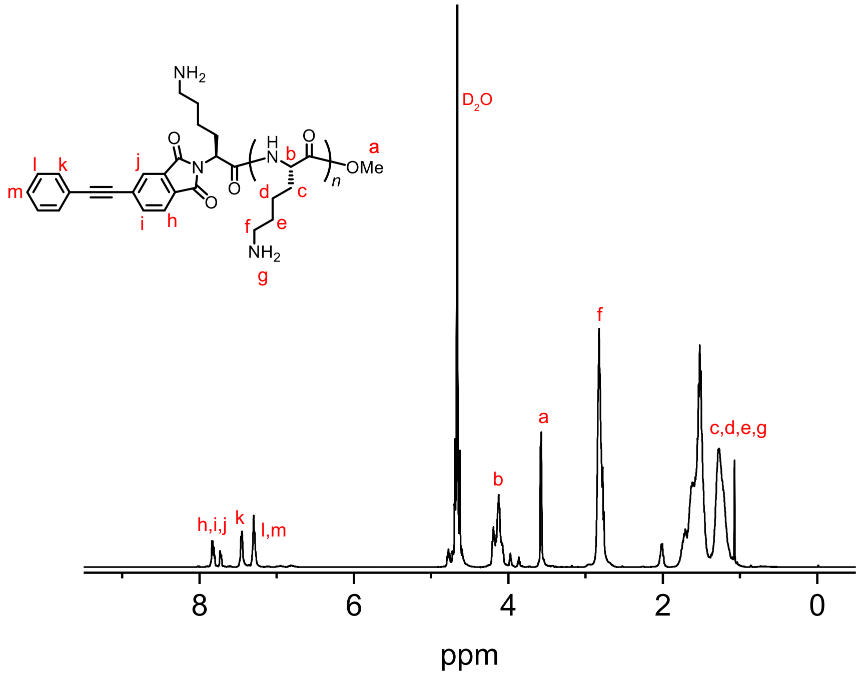

Fig. S1 ^{1}H NMR spectrum of PhC≡CPh-pLys (**1**) in D_2O.

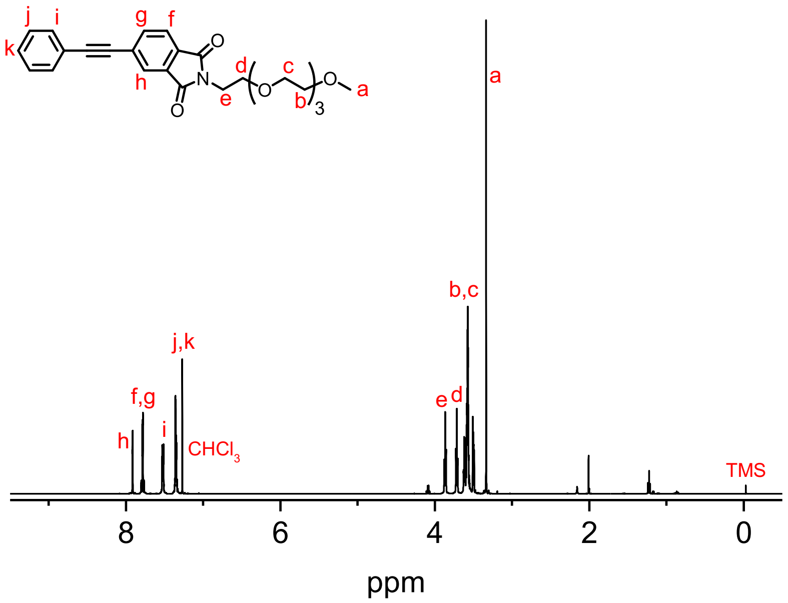

Fig. S2 ^{1}H NMR spectrum of PhC≡CPh-PEG (**2**) in $CDCl_3$.

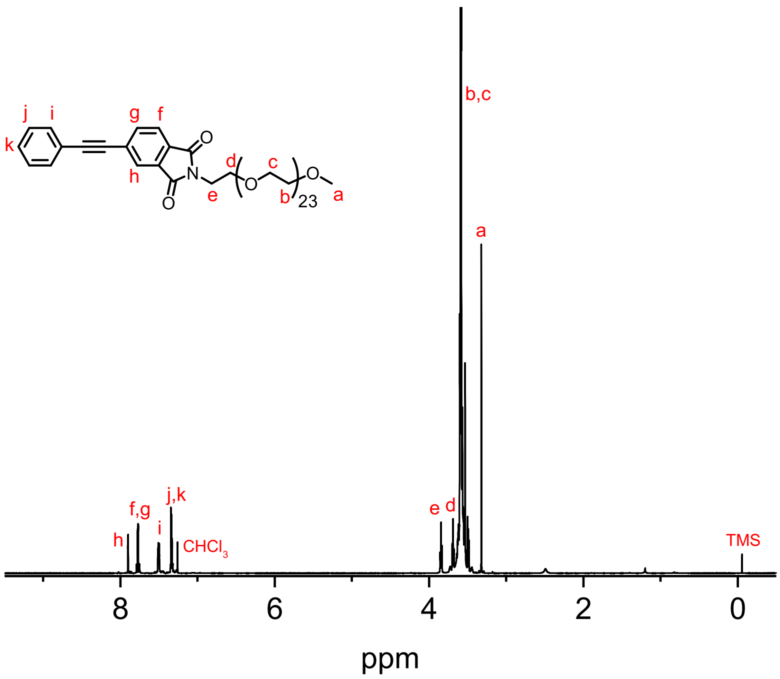

Fig. S3 ^{1}H NMR spectrum of PhC≡CPh-PEG (**3**) in $CDCl_3$.

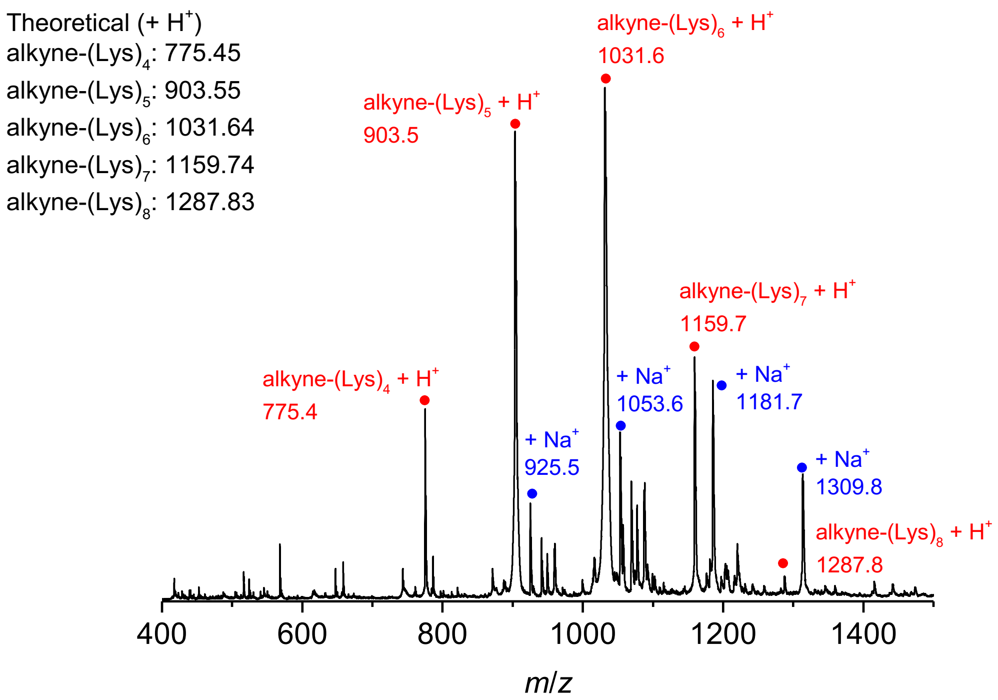

Fig. S4 MALDI-TOF MS spectrum of PhC≡CPh-pLys (**1**).

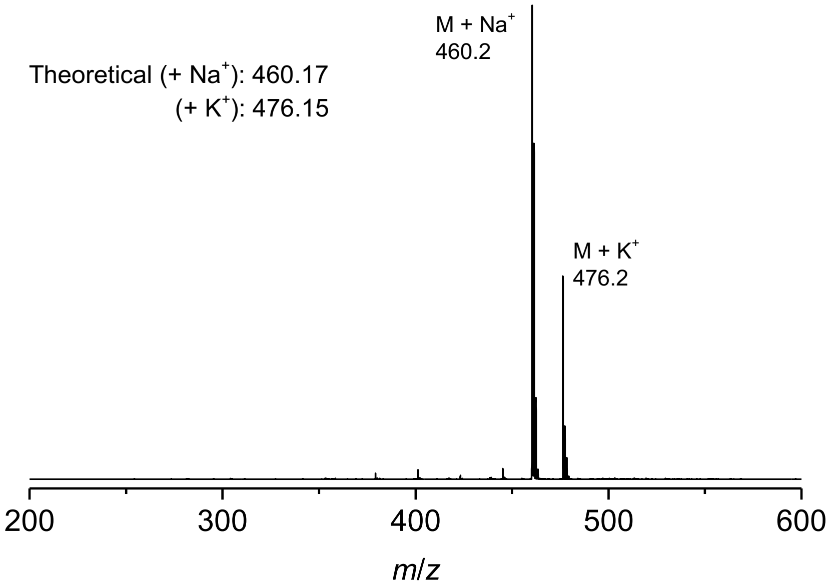

Fig. S5 MALDI-TOF MS spectrum of PhC≡CPh-TEG (**2**).

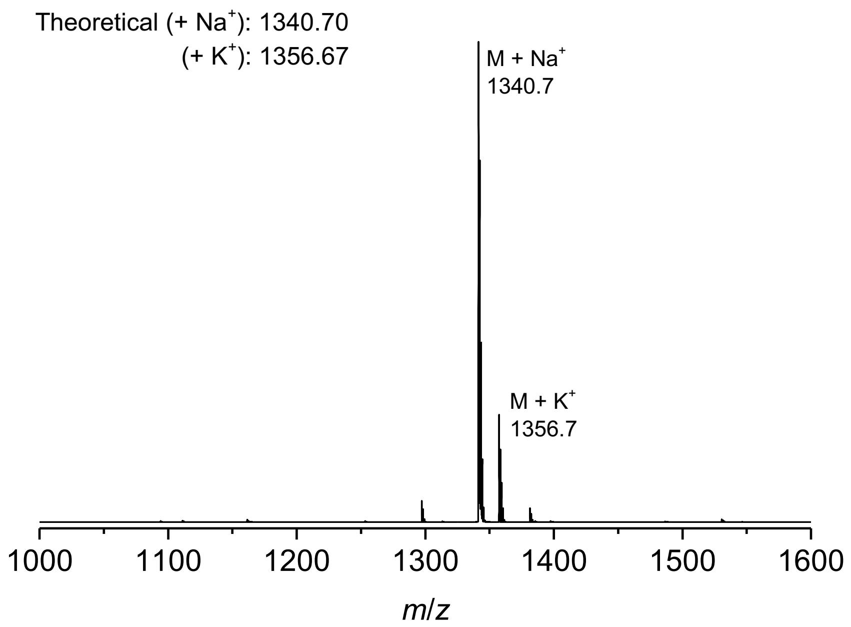

Fig. S6 MALDI-TOF MS spectrum of PhC≡CPh-PEG (**3**).

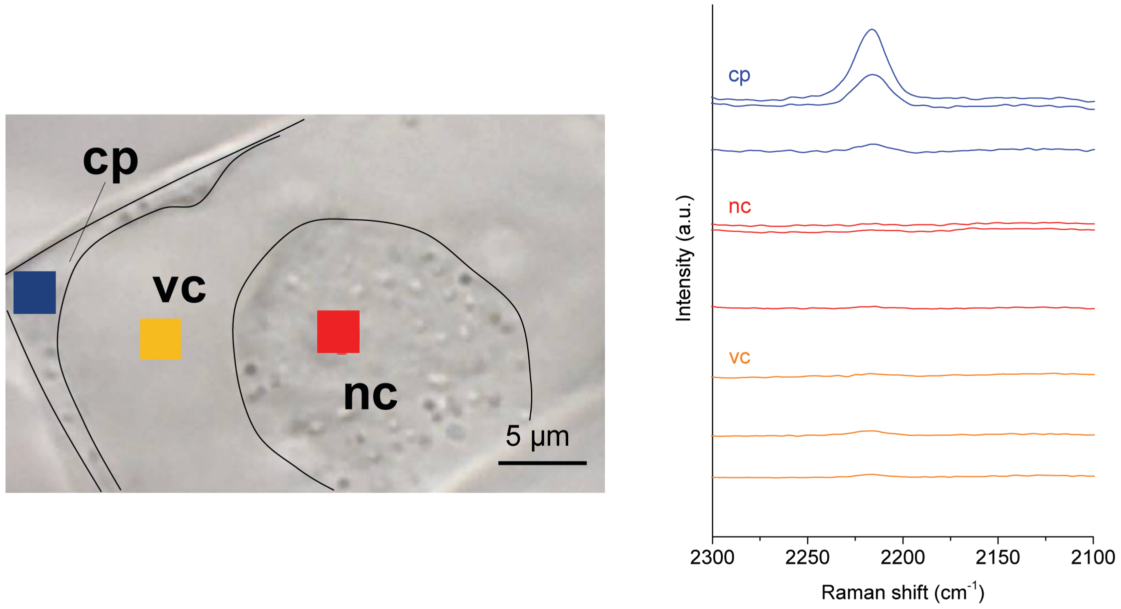

Fig. S7 Raman spectra on various cites of a BY-2 cell treated with PhC≡CPh-pLys (**1**). Three spectra were obtained at different positions in each cite (cp: cytoplasm, nc: nucleus, vc: vacuole).

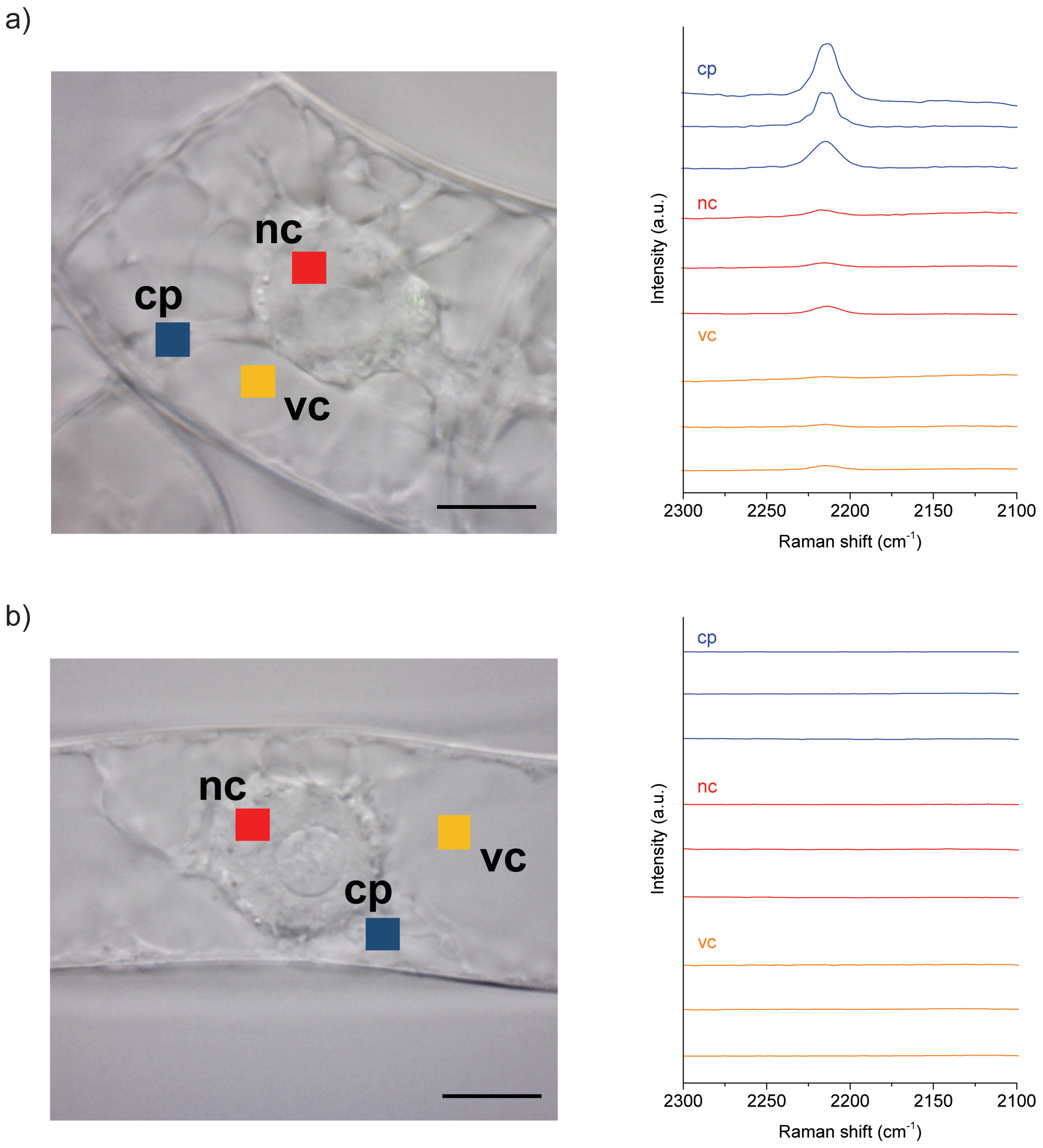

Fig. S8 Raman spectra on various cites of a BY-2 cell treated with (a) PhC≡CPh-TEG (**2**) and (b) PhC≡CPh-PEG (**2**). Three spectra were obtained at different positions in each cite (cp: cytoplasm, nc: nucleus, vc: vacuole). Scale bars are 5 μm.

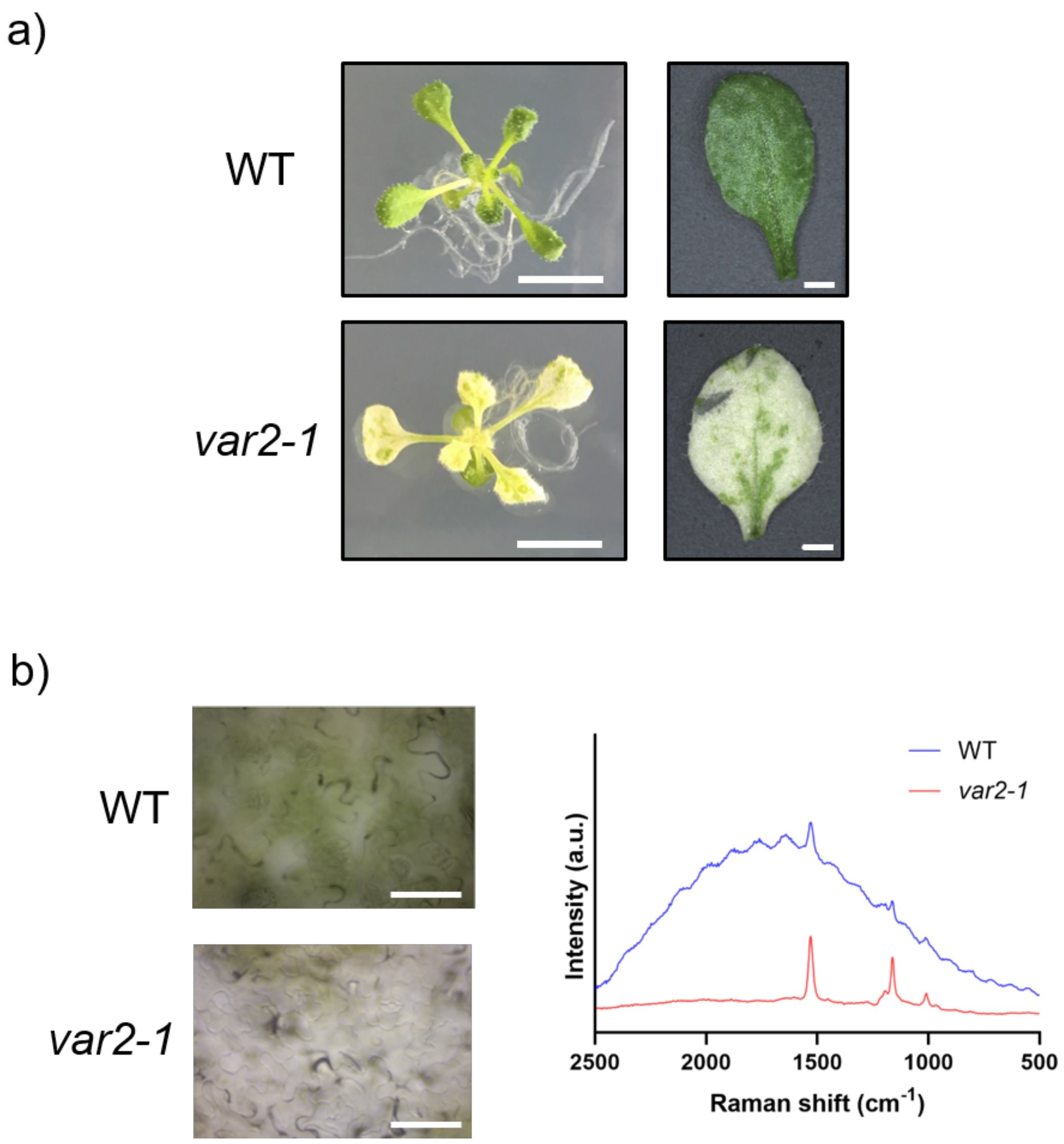

Fig. S9 Phenotypic comparison between wild-type (WT) and *var2-1* mutant. (a) Morphology of *var2-1* mutant plant. The scale bars indicate 1 cm or 1 mm, respectively. (b) Raman spectrum in WT and *var2-1* leaf cells. The Raman signal of carotenoid (1005 cm^{-1}, 1158 cm^{-1}, and 1525 cm^{-1}) was clearly detected in the *var2-1* mutant plant. In WT, due to raising the baseline by chlorophyll-derived autofluorescence in the chloroplast, obscuring the carotenoid signals. The scale bars indicate 50 μm.

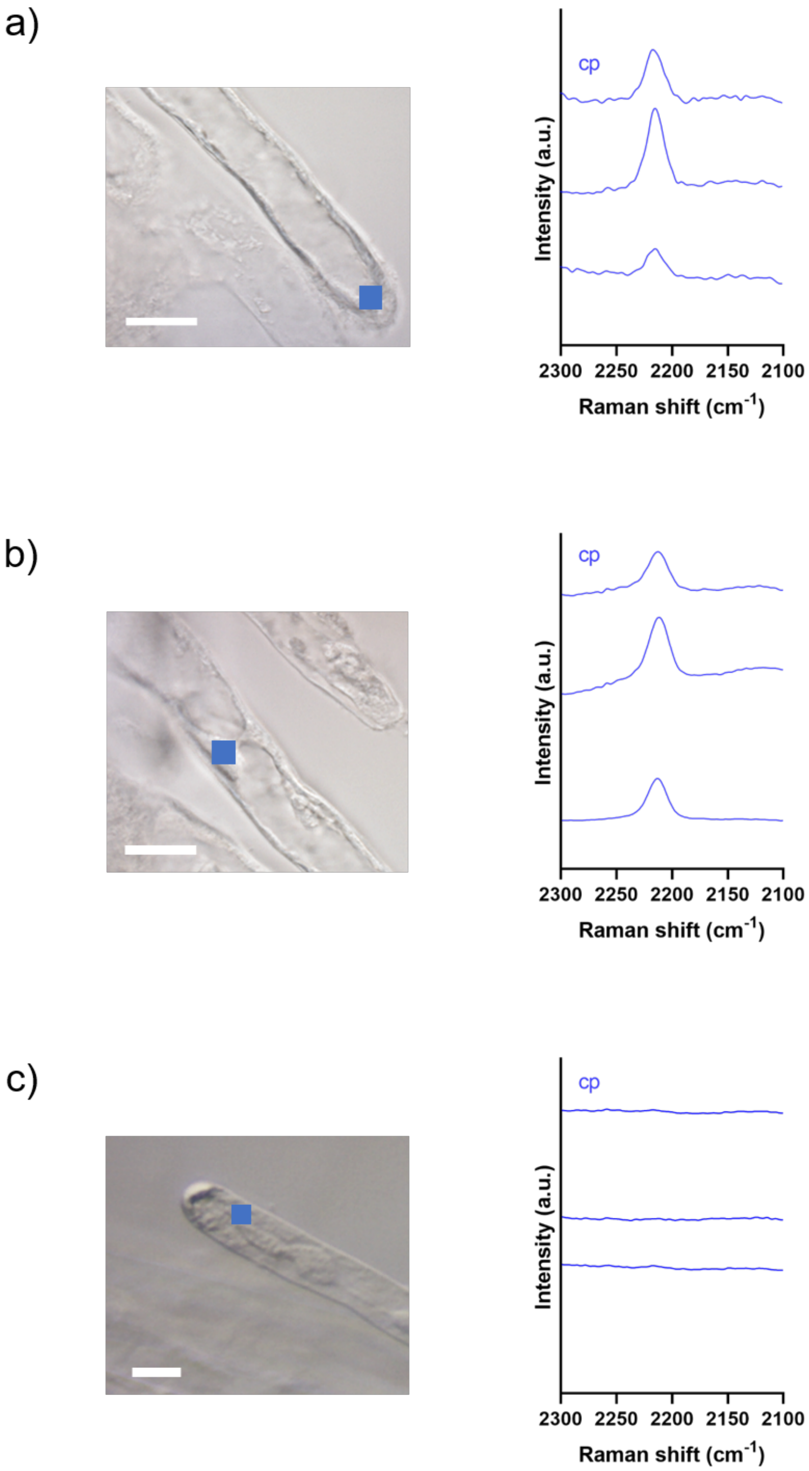

Fig. S10 Raman spectra on various cites of a *var2-1* root cell treated with (a) PhC≡CPh-pLys (**1**), (b) PhC≡CPh-TEG (**2**) and (c) PhC≡CPh-PEG (**3**). Three spectra were obtained at different positions in each cite (cp: cytoplasm). Scale bars are 10 μm.

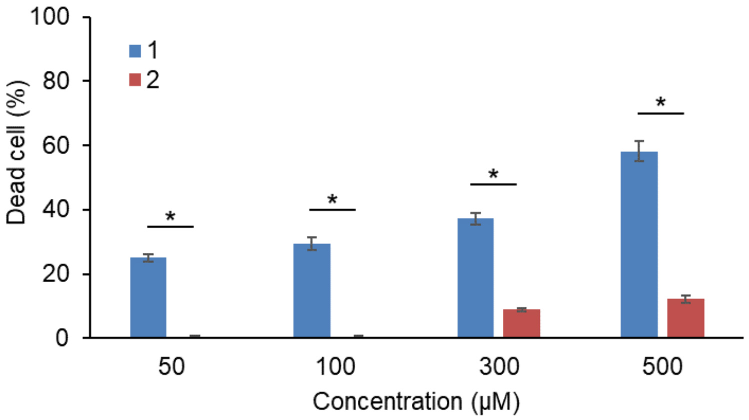

Fig. S11 Cell viability assay with BY-2 cells. After BY-2 cells were incubated with PhC≡CPh-pLys (**1**) or PhC≡CPh-TEG (**2**) for 2 h at concentrations ranging from 50 to 500 μM, the dead cell ratio was calculated by dividing the OD_{600} value by that of "100% dead cells" which were treated at 100°C for 1 h (n = 3, *p < 0.01, *t*-test, mean±SD).

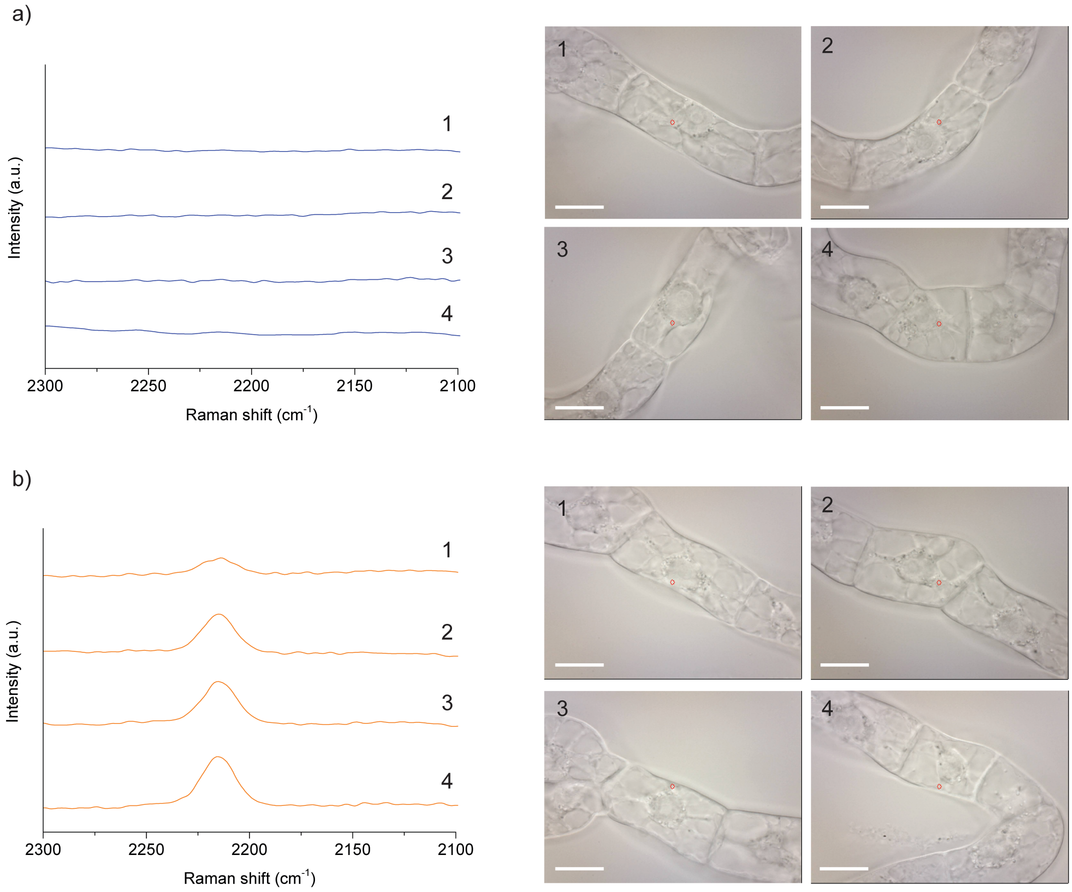

Fig. S12 Raman spectra on BY-2 cells treated with (a) PhC≡CPh-pLys (**1**) and (b) PhC≡CPh-TEG (**2**) incubated at 4 °C for 5 h. Scale bars are 10 µm.

a)

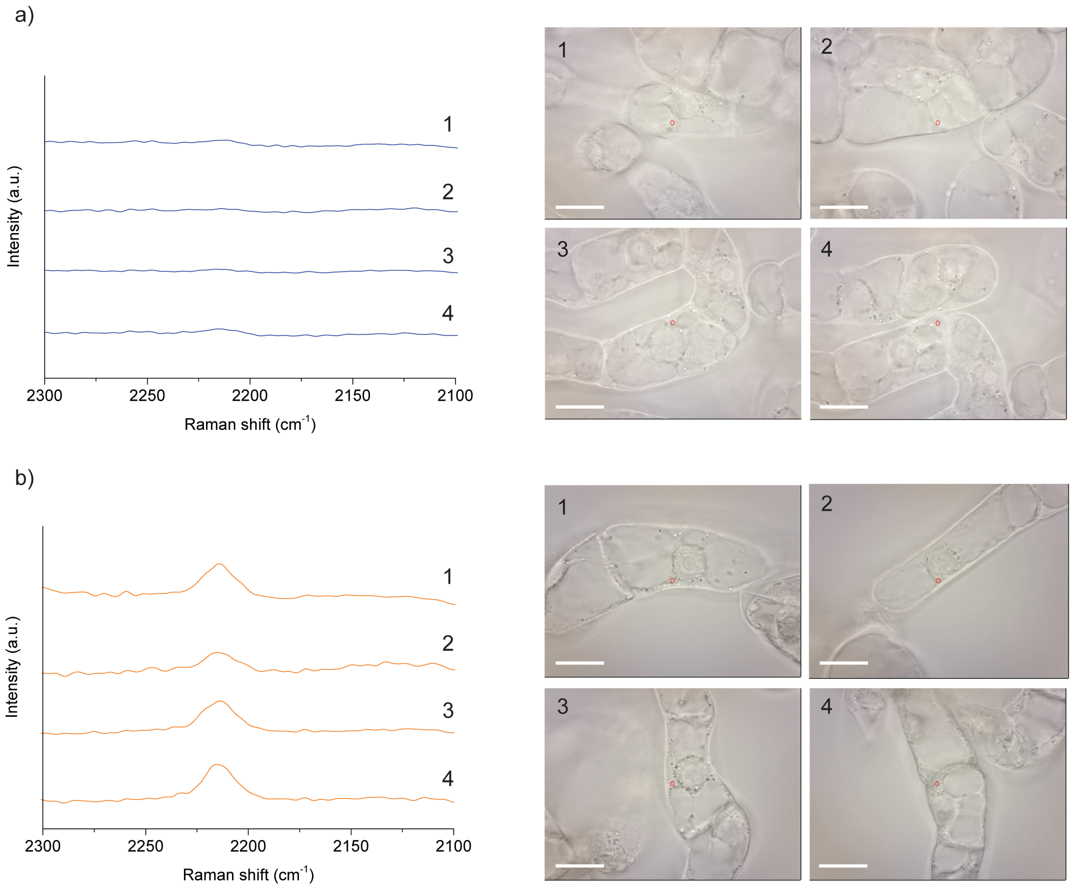

Fig. S13 Raman spectra on BY-2 cells treated with (a) PhC≡CPh-pLys (**1**) and (b) PhC≡CPh-TEG (**2**) incubated at 26 °C for 5 h in the presence of 40 µM Wortmannin. Scale bars are 10 µm.

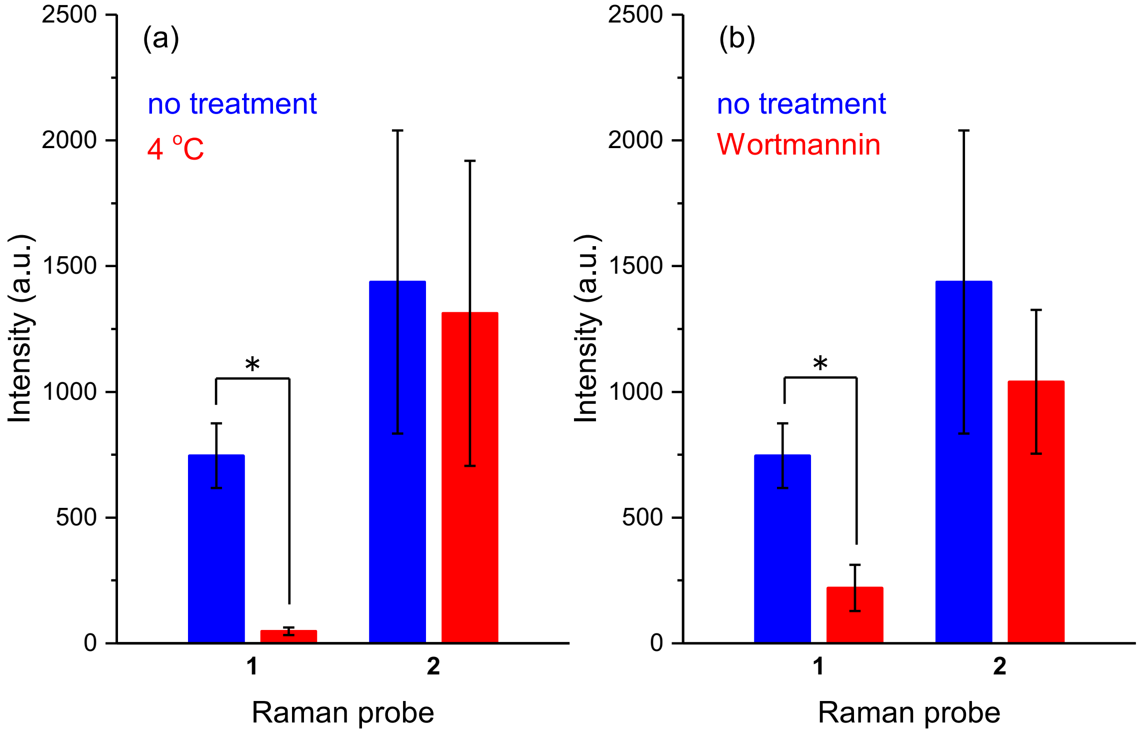

Fig. S14 Raman intensity at 2215 cm^{-1} in the Raman spectra obtained for BY-2 cells treated with PhC≡CPh-pLys (**1**) or PhC≡CPh-TEG (**2**) incubated at (a) 4 °C for 5 h and (b) 26 °C for 5 h in the presence of 40 μM Wortmannin (n = 4, *p < 0.05, *t*-test, mean±SD).

References

1. T. Miyamoto, K. Tsuchiya and K. Numata, *Biomacromolecules*, 2019, **20**, 653-661.
2. Y. Kato, E. Miura, K. Ido, K. Ifuku and W. Sakamoto, *Plant Physiology*, 2009, **151**, 1790-1801.
3. Y. Ozeki, W. Umemura, Y. Otsuka, S. Satoh, H. Hashimoto, K. Sumimura, N. Nishizawa, K. Fukui and K. Itoh, *Nat. Photonics*, 2012, **6**, 845.
4. K. Numata, Y. Horii, K. Oikawa, Y. Miyagi, T. Demura and M. Ohtani, *Scientific Reports*, 2018, **8**, 10966.
